# The impact of *HBB*‐related hemoglobinopathies carrier status on fetal fraction in noninvasive prenatal screening

**DOI:** 10.1002/pd.6127

**Published:** 2022-03-23

**Authors:** Manesha Putra, Kristjan Eerik Kaseniit, Melissa A. Hicks, Dale Muzzey, David Hackney

**Affiliations:** ^1^ Department of Obstetrics and Gynecology, Division of Maternal‐Fetal Medicine University Hospital Cleveland Medical Center Cleveland Ohio USA; ^2^ Department of Obstetrics and Gynecology, Division of Maternal‐Fetal Medicine Metro Health Medical Center Cleveland Ohio USA; ^3^ Department of Obstetrics and Gynecology, Division of Maternal‐Fetal Medicine Case Western Reserve University Cleveland Ohio USA; ^4^ Research & Development Myriad Genetics, Inc. Salt Lake City Utah USA; ^5^ Department of Pathology Detroit Medical Center University Laboratories Detroit Michigan USA

## Abstract

**Objective:**

We evaluated whether there is an association between β‐globin (*HBB*) pathogenic variants and fetal fraction (FF), and whether the association has a clinically relevant impact on non‐invasive prenatal screening (NIPS).

**Method:**

A whole‐genome sequencing NIPS laboratory database was retrospectively queried for women who underwent NIPS and carrier screening of both *HBB* and the α‐globin genes (*HBA1/HBA2*). Women affected with either condition were excluded from the study, yielding a cohort size of 15,853. A “corrected FF” was obtained via multivariable linear regression adjusted for the systematic impacts of maternal age, gestational age and BMI. Corrected FF distributions of *HBB* and *HBA1/HBA2* carriers were each compared to non‐carriers using the Kolmogorov‐Smirnov test.

**Results:**

In this cohort, 291 women were carriers for *HBB* alone*,* and 1016 were carriers for *HBA1/HBA2* alone. The *HBB* carriers had a lower corrected FF when compared to non‐carriers (*p* < 0.0001). There was no difference in corrected FF among carriers and non‐carriers of *HBA1/HBA2*.

**Conclusion:**

Carriers of pathogenic variants in the *HBB* gene, but not the *HBA1/HBA2* genes, are more likely to have lower FF when compared to women with structurally normal hemoglobin. This decrease in FF could result in an elevated test‐failure rate if FF thresholds were used.

## Key points

What is already known about this topic?

- Multiple factors affect fetal fraction during noninvasive prenatal screening (NIPS), such as maternal BMI and gestational age. Patients affected with Hb beta chain‐related hemoglobinopathies have been shown to have lower fetal fraction during NIPS.

What does this study add?

- This study demonstrates that *HBB* carriers—not just affected patients—also have lower fetal fraction on NIPS, adding to the literature of factors that could affect fetal fraction and NIPS performance.

## 1 INTRODUCTION

The American College of Obstetricians and Gynecologists (ACOG) has long recommended that aneuploidy screening be offered to all pregnant women regardless of maternal age.^1^ Since its commercial availability, non‐invasive prenatal screening (NIPS) has been rapidly adopted in both high‐ and low‐risk settings.^2^, ^3^ Despite its high sensitivity and specificity as a screening test, NIPS has its own limitations: many laboratories fail samples because of an insufficient fraction of trophoblastic (“fetal”) circulating cell‐free fetal DNA (cffDNA) in maternal plasma.^4^, ^5^

Fetal fraction (FF) is one of the quality metrics in NIPS that directly affects test performance because it reflects the actual proportion of cffDNA to the total circulating cell‐free DNA.^6^ Lower FF has been shown to reduce the detection rate for common aneuploidies to as low as 62% in some reports, however it has also been recently shown that NIPS can have high accuracy across the spectrum of FF values.^7^, ^8^ Various clinical factors such as fetal aneuploidy, maternal weight and gestational age have been shown to affect FF.^9^, ^10^ We also previously reported the association of clinically significant beta‐chain hemoglobin gene (*HBB*)*‐*related hemoglobinopathies, especially sickle cell anemia, and low FF.^11^ We speculated that this association could be due to an increase in maternal cell necrosis (from increased sickling or chronic anemia) causing a dilutional effect on FF.

*HBB*‐hemoglobinopathy carrier status is typically considered benign as carriers do not usually experience conspicuous clinical manifestations. However, in rare cases they could present with mild‐moderate clinical manifestations.^12^, ^13^, ^14^, ^15^ Because of this, we hypothesized that carriers of *HBB*‐hemoglobinopathies may also have some degree of increased maternal cell necrosis causing reduction in FF. We sought to evaluate whether there is an association between *HBB* pathogenic variants carrier status and FF, and whether it would be expected to have a clinically relevant impact on NIPS.

## 2 MATERIALS AND METHODS

This is a retrospective cohort study. This research is approved by University Hospital Cleveland Medical Center Institutional Review Board, and the analysis of de‐identified patients was deemed exempt by Western Institutional Review Board. The database of a laboratory that performs both whole‐genome sequencing (WGS)‐based NIPS and sequencing‐based carrier screening (Myriad Women's Health, South San Francisco, CA) was retrospectively queried from 2016 to 2019 to assemble the study cohorts. Large‐scale clinical experience with this NIPS and its fetal‐fraction methodology were characterized previously.^8^ All women included in the cohorts had previously consented to de‐identified studies.

The first cohort consisted of all women with singleton pregnancies who underwent NIPS who were found to be carriers for an *HBB*‐hemoglobinopathy, including beta thalassemia trait and other heterozygous pathogenic variants in *HBB* (e.g., Hemoglobin C, D and E traits). To assess the impact of mild anemia, we assembled a second cohort of women with singleton pregnancies who underwent NIPS and were found to have two or three copies of the *HBA1/HBA2* genes (i.e., alpha thalassemia silent carriers and alpha thalassemia trait carriers). All women whose carrier screening results suggest that they are themselves clinically affected by *HBB* or *HBA1/HBA2* hemoglobinopathies were excluded as they are expected to have a more severe phenotype. To reduce potential confounding from aneuploid results, we also excluded all women who received screen‐positive results for any fetal chromosome abnormality on NIPS because aneuploidy has been shown to affect fetal fraction.^9^ Concurrent carriers for both *HBB* and *HBA1/HBA2* hemoglobinopathies were also removed from the analysis. Both of these cohorts were then compared to a control group of women who had low‐risk NIPS results (i.e., negative for trisomy 21, 18, 13 and sex chromosome aneuploidies) and were not carriers for any pathogenic variants in *HBB* or *HBA1/HBA2.* A subgroup analysis was performed among women with sickle cell trait (a subgroup of *HBB‐*hemoglobinopathy carriers) because our previous study showed larger impact size among this group.^11^

Multivariable linear regression was used to adjust for the systematic impacts of maternal age, gestational age and BMI to yield a “corrected FF” for each patient. Regression coefficients were 0.01% for each year of maternal age, 0.32% for each week of gestational age, and −0.27% for each unit of BMI. We elected to not include race and ethnicity in the regression due to a report of the varying impact of these factors.^16^ The significance of comparisons among cohorts and control groups was assessed by using the Kolmogorov‐Smirnov test on the respective corrected FF distributions.

The clinical test‐failure rate represents the number of test failures divided by the number of total tests performed. The laboratory used in this study does not use a fixed FF cut‐off to determine test‐failure, rather it employs multiple quality‐control metrics to make the calls. However, FF cutoffs are still commonly used by many vendors. To assess the hypothetical clinical impact of the shift in FF distributions, we calculated the expected test‐failure rate for a given cohort by integrating the area under the curve for a beta distribution fit to the cohort's corrected FF values (the beta distribution has been shown previously and herein to be a good model for the FF distribution).^17^

## 3 RESULTS

**FIGURE 1:**
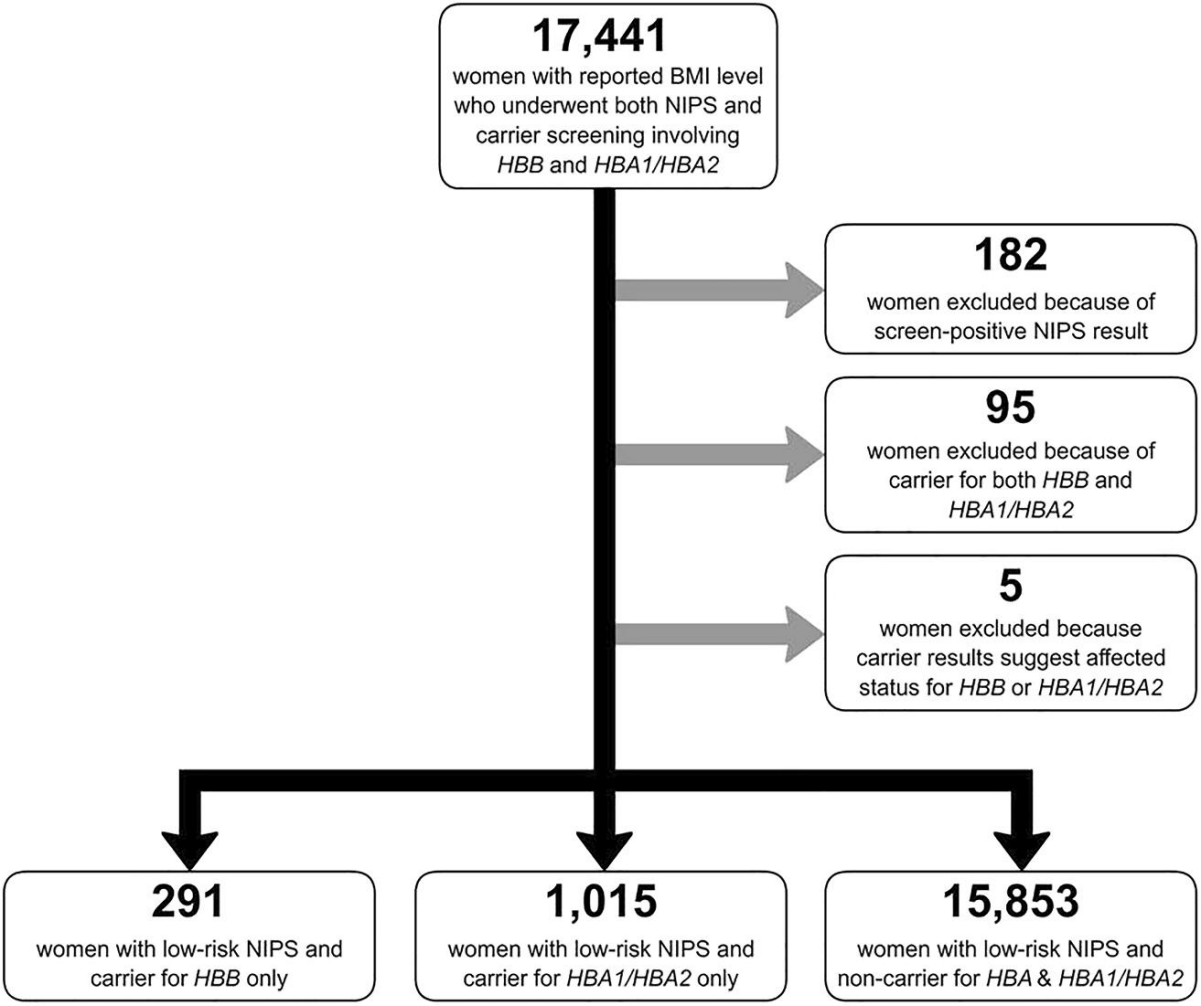
Representation of cohort size, exclusion criteria, and carrier status

Among women who had low‐risk screening results on NIPS and underwent carrier screening involving *HBB* and *HBA1/HBA2*, 17,159 were either non‐carriers for both conditions or carriers for only one of the conditions (Figure 1). A total of 291 women were *HBB* carriers only, and 1015 were *HBA1/HBA2* carriers only. As a comparison group, 15,853 women were found to be non‐carriers for either condition. Demographic characteristics of *HBB* carriers and non‐carriers are included in Table 1.

**TABLE 1:** Demographic characteristics of study groups

Characteristic	Non‐carriers of *HBB* and *HBA* (*N* = 15,853)	*HBB* carriers (*N* = 291)	HbS carriers (*N* = 118)	*HBA1/HBA2* carriers (*N* = 1015)
Maternal age	32.3 [29–36]	31.7 [27–37]	30.3 [24–36]	31.5 [27–36]
BMI	26.5 [22.2–29.5]	27.4 [23.0–30.1]	29.1 [24.5–32.5]	27.8 [22.9–31.6]
GA	12.8 [10.9–13.1]	13.6 [11.4–14.1]	14.0 [12.0–14.4]	13.6 [11.3–13.9]
FF	9.1% [6.5%–11.2%]	8.3% [6.1%–9.9%]	8.1% [5.8%–9.6%]	9.1% [6.5%–10.9%]
% African	5.3%	33.3%	56.8%	40.0%
% Asian	10.8%	19.9%	0.9%	14.6%
% non‐Asian and non‐African	83.9%	46.8%	42.3%	45.4%

*Note*: For maternal age, body mass index, gestational age, and fetal fraction, the displayed values are the mean and interquartile range.

Abbreviations: BMI, body mass index; FF, fetal fraction; GA, gestational age; HbS carriers, sickle cell trait.

**FIGURE 2:**
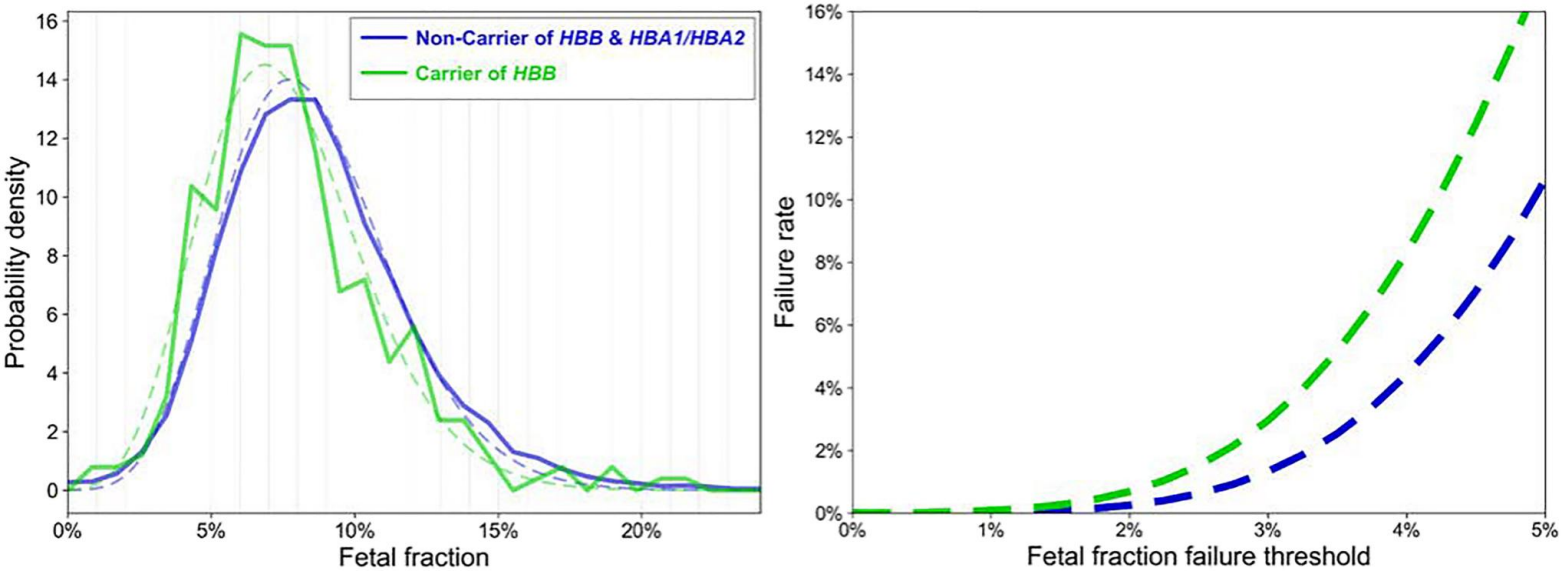
(A) Probability density across fetal fraction levels in *HBB* carrier versus non‐carrier (solid line: actual data, dashed line: fitted data). (B) Expected test‐failure rate (*y*‐axis) due to sub‐threshold fetal fraction (FF) in *HBB* carrier versus non‐carrier with varying FF failure threshold (*x*‐axis)

Uncorrected FF was lower among *HBB* carriers relative to non‐carriers (*p* < 0.0001), and this difference persisted after the FF was corrected for maternal age, gestational age and BMI (*p* < 0.0001, Figure 2A). Clinically, no samples were failed in this cohort due to low FF. The expected test failure rate among *HBB* carrier women using a FF cutoff of 4% was 8.3%, as compared to an expected 4.4% rate in non‐carriers (Figure 2B).

**FIGURE 3:**
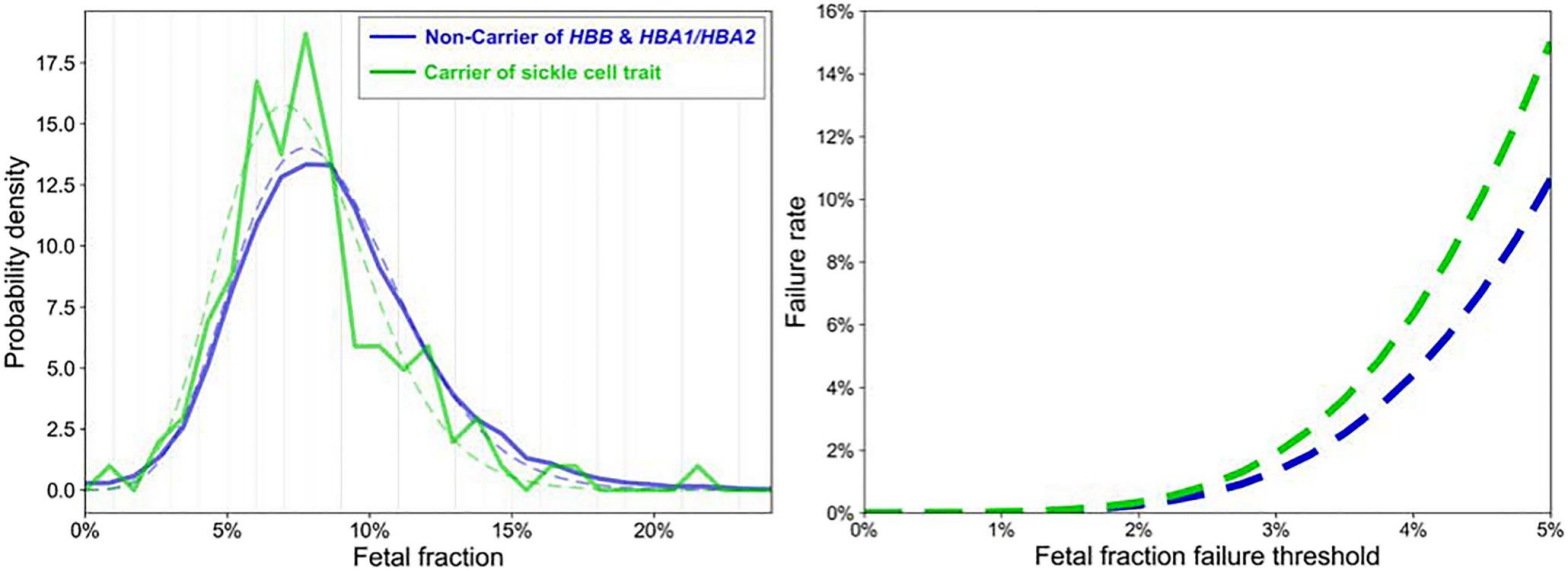
(A) Probability density across fetal fraction levels in sickle‐cell trait carriers (HbS) versus non‐carrier (solid line: actual data, dashed line: fitted data). (B) Expected test‐failure rate (*y*‐axis) due to sub‐threshold fetal fraction (FF) in sickle‐cell trait carrier versus non‐carrier with varying FF failure threshold (*x*‐axis)

In the planned subgroup analysis, 118 women were found to have sickle cell trait (demographic characteristics in Table 1). The uncorrected FF of sickle cell trait carriers was lower compared to non‐carriers (*p* < 0.003). After the FF was corrected for maternal age, gestational age and BMI, the sickle cell trait carrier group had lower corrected FF when compared to the non‐carrier group (*p* < 0.0161; Figure 3A). The expected test‐failure rate among women with sickle cell trait was 6.3% using a FF cutoff of 4% (Figure 3B).

**FIGURE 4:**
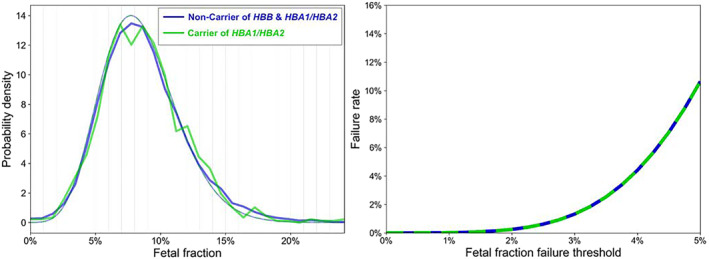
(A) Probability density across fetal fraction levels in *HBA1*/*HBA2* carriers versus non‐carrier (solid line: actual data, dashed line: fitted data). (B) Expected test‐failure rate (*y*‐axis) due to sub‐threshold fetal fraction (FF) in *HBA1*/*HBA2* carriers versus non‐carrier with varying FF failure threshold (*x*‐axis)

In the second cohort, 1015 women were identified to be carriers of *HBA1/HBA2* pathogenic variants (demographic data in Table 1). After the FF was corrected for maternal age, gestational age and BMI, the FF values did not differ among carrier and non‐carrier groups (*p* > 0.05; Figure 4A; uncorrected FF also did not vary significantly, *p* > 0.05). The expected test‐failure rate (4.4%) at a 4% FF cutoff was the same among carriers and non‐carriers of *HBA1/HBA2* (Figure 4B).

## 4 DISCUSSION

### 4.1 Principal findings

Carriers of pathogenic variants in the *HBB* gene are more likely to have lower FF when compared to non‐carriers. This decrease in FF yields more low‐FF samples (i.e., FF < 4%) and could result in higher no‐call rate if an FF threshold is used. In the subgroup analysis of women with sickle cell trait, we also observed lower FF when compared to non‐carriers. These observations were similar to previous findings in women who were affected by *HBB‐*related hemoglobinopathies, albeit our current study has a smaller effect size.^11^ This lends support to our hypothesis that carriers of pathogenic variants in *HBB* have a subclinical increase of maternal cell necrosis that leads to a dilutional effect on FF. By contrast, *HBA1/HBA2* pathogenic variant carrier status is not associated with lower FF when compared to non‐carriers. Though women who were carriers of both *HBB* and *HBA1/HBA2* were excluded from the main analysis to allow for disambiguation of the respective effects of each gene, we found that they also had significantly lower FF than non‐carriers (8.6% mean FF in carriers of both genes vs. 9.1% mean FF in non‐carriers; *p* < 0.05).

### 4.2 Clinical implications

While the reduction in FF associated with being a carrier for *HBB‐*related hemoglobinopathies is smaller than previously observed in women fully affected by *HBB*‐related hemoglobinopathies, the impact on FF could still be appreciable^11^: aside from increasing the number of samples at low FF and possibly increasing test failures, a reduction in FF could also theoretically reduce the test performance on certain NIPS platforms.^7^, ^18^ More importantly, the impact of FF reduction associated with being a carrier for *HBB‐*related hemoglobinopathies could be particularly acute as NIPS evolves to explore genome‐wide microdeletions or single‐gene fetal anomalies. Because these NIPS strategies interrogate regions far smaller than whole chromosomes, we anticipate a higher impact on test performance for these types of assays. Importantly, emerging technologies that increase the FF of samples undergoing NIPS would be expected to mitigate the impact of downward pressure on FF in *HBB* carriers: preferentially selecting DNA fragments smaller than 160 base pairs has been shown to be an effective method to significantly increase FF and improve test performance.^19^, ^20^, ^21^ Nevertheless, we suggest discussing the potential impact of *HBB‐*related hemoglobinopathies carrier status in pre‐ and post‐test counseling for women who desire NIPS for aneuploidy screening.

### 4.3 Research implication

Characterization of the actual biological mechanism that underlies the association between lower FF and being a carrier of *HBB‐*related hemoglobinopathies is of great interest but outside the scope of this study. We speculate that the presence of subclinical vasculopathies in this group causes a higher proportion of maternal fraction and ultimately lower fetal fraction. Further studies could be relevant even beyond the field of prenatal diagnosis. If our hypothesis regarding a subclinical increase in maternal vasculopathies is verified, it is possible that *HBB‐*related hemoglobinopathies carrier status could also affect test performance of cell‐free DNA‐based cancer diagnostics.

### 4.4 Strengths and limitations

While we controlled for some factors that could be associated with changes in FF, this is a retrospective study with an inherent limitation for availability of complete clinical data. We were not able to control for all previous factors that have been associated with changes in FF, such as placental or maternal diseases. It is possible that other factors not previously known affect FF particularly among *HBB‐*related hemoglobinopathy carriers. We also do not know the status of the fetuses with respect to hemoglobinopathies; as such we cannot comment on the impact of this variable to FF, if any. Nevertheless, this is the first study to evaluate the association between FF and carrier status for *HBB‐*related hemoglobinopathies. We used a large database of women with NIPS and carrier screening to reduce selection bias, enabling us to exclude women who received aneuploid NIPS results or were concurrent carriers for both *HBB* and *HBA1/HBA2*.

## 5 CONCLUSIONS

Our study joins the rich literature of many factors that affect FF. *HBB‐*related hemoglobinopathy carrier status should be considered in counseling for women who are interested in NIPS as a method for aneuploidy screening, especially when coverage beyond common aneuploidies is desired. Further understanding of this topic could be crucial as NIPS utilization is expanded beyond common aneuploidies.

## CONFLICT OF INTEREST

Authors Kristjan Eerik Kaseniit and Dale Muzzey are employed by Myriad, Inc, a company that performs non‐invasive prenatal screening and expanded carrier screening. Authors Manesha Putra, Melissa A. Hicks and David Hackney declare no conflict of interest.

## Data Availability

The data that support the findings of this study are available on request from the corresponding authors.
